# Supplementary material for: HCS-Splice: A High-Content Screening Method to Advance the Discovery of RNA Splicing-Modulating Therapeutics
Source: Cells. 2023 Jul 28;12(15):1959. doi: 10.3390/cells12151959 (PMC10417277; doi:10.3390/cells12151959)
Supplement: Supplementary file 1 [file cells-12-01959-s001.zip › cells-2496692-supplementary.pdf]

Supplemental Figures and Legend

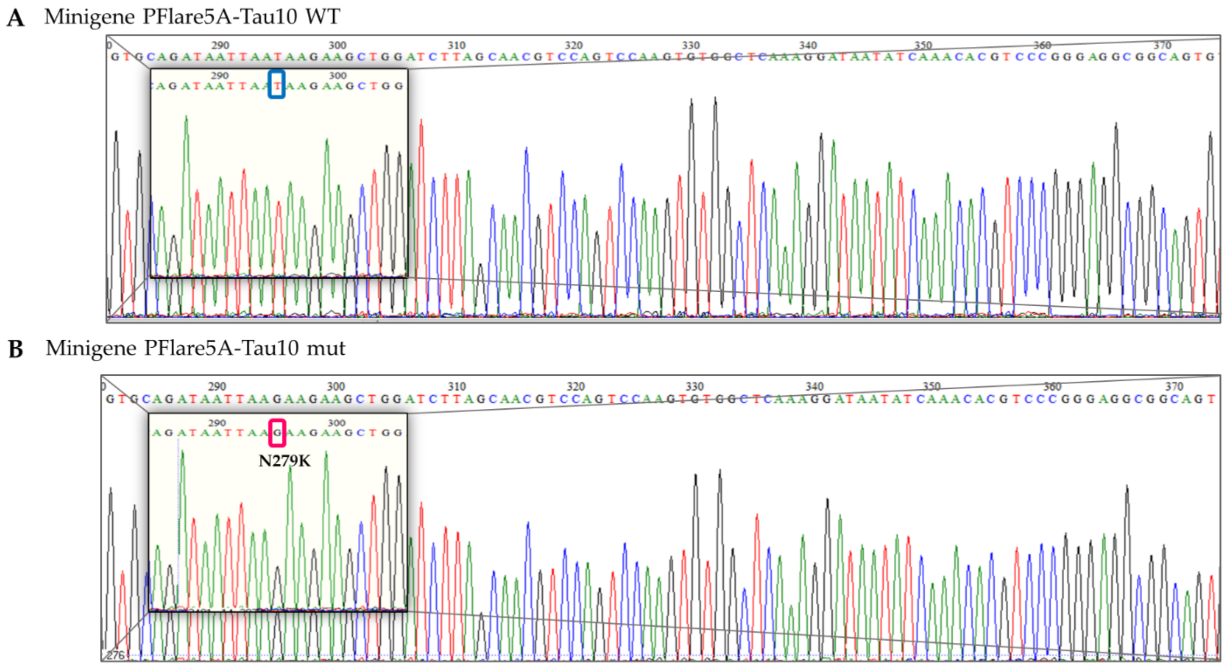

Figure S1. Chromatogram sequence. (a) of PFlare5A-Tau10 WT reporter plasmid (Minigene PFlare5A-Tau10 WT) and **(b)** of PFlare5A-Tau10 mut reporter plasmid (Minigene PFlare5A-Tau10 mut). Chromatogram shows the correct position of N279K point mutation in the PFlare5A-Tau10 mut reporter plasmid (BMR genomics, Padova, Italy).

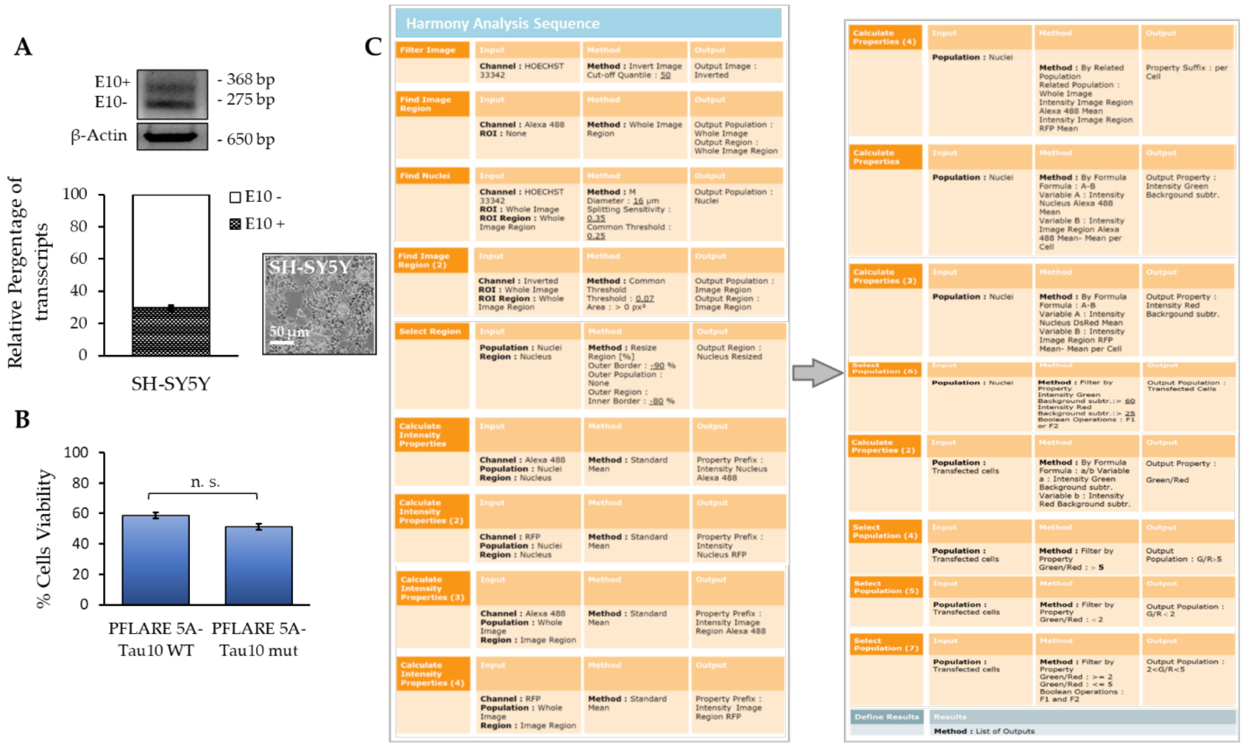

Figure S2. SH-SY5Y cell lines analysis and overall HCS-splice protocol procedure. (a) Semiquantitative RT-PCR of Exon 10 endogenous expression levels in the SH-SY5Y cell line was performed as described in the material and methods section. β actin was used as a housekeeping gene (650 bp), and values were represented as mean ± SD (n=3). **(b)** Percentage of cell viability in SH-SY5Y cells after transfection (48 hours) was measured by the Trypan Blue assay. Data represent mean ± SEM obtained from triplicates. **(c)** Representative steps of the Harmony analysis protocol workflow used for the HCS-splice methods.

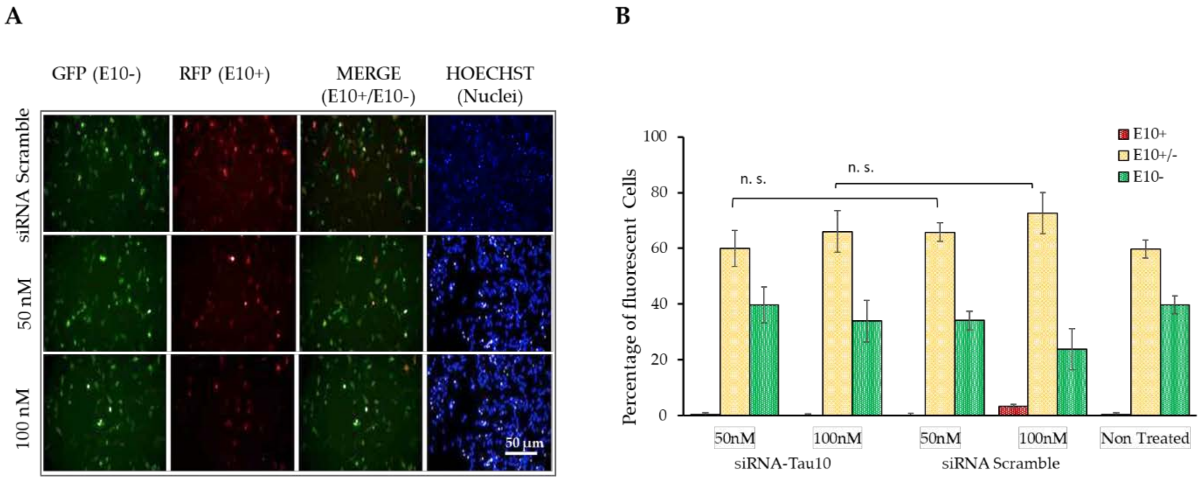

Figure S3. Exon 10 expression of PFlare5A-Tau10 WT report plasmid after treatment with siRNA-Tau10. **(a)** Representative images of SH-SY5Y cell line cotransfected with Minigene PFlare5A-Tau10 WT and treated with siRNA-Tau10 at concentrations of 50 and 100 nM (Top to bottom). The cells were acquired by a High-Content screening system (Operetta, *PerkinElmer*) after 48 hours of treatments, as described in Figure 2. The scale bar represents 50 µm. **(b)** The histogram represents the relative percentages of the three sub-populations of cells containing the reporter treated respectively with siRNA-Tau10 and Scramble siRNA control at a concentration of 50 nM and 100 nM. The values represent mean ± SD (n=3). Asterisks (*) indicate significant differences (t-test, n.s. P > 0.05, *P ≤ 0.05, **P ≤ 0.01, ***P ≤ 0.001, **** P ≤ 0.0001).
